# Grade G2 Rectal Neuroendocrine Tumor Is Much More Invasive Compared With G1 Tumor

**DOI:** 10.3389/fonc.2021.646536

**Published:** 2021-03-11

**Authors:** Yi-Wei Li, Yi-Ping He, Fang-Qi Liu, Jun-Jie Peng, San-Jun Cai, Ye Xu, Ming-He Wang

**Affiliations:** ^1^ Department of Colorectal Surgery, Fudan University Shanghai Cancer Center, Shanghai, China; ^2^ Department of Oncology, Shanghai Medical College, Fudan University, Shanghai, China; ^3^ Department of Endoscopy, Fudan University Shanghai Cancer Center, Shanghai, China

**Keywords:** neuroendocrine tumor (NET), neuroendocrine *neoplasm* (NEN), carcinoid, metastasis, treatment

## Abstract

**Background:**

To compare clinicopathologic feature of rectal neuroendocrine tumor (NET) grade G1 with G2 NET.

**Methods:**

Six hundred-one cases of rectal G1 and G2 NETs diagnosed in our center were analyzed.

**Results:**

Of 601 cases of rectal NET, 515 cases were with grade G1 and 86 cases were with grade G2. Median tumor size was 0.7 cm. Compared with G1 NET, G2 tumors were with significantly larger tumor size (0.8 vs 2.2 cm, p < 0.001), less percentages of patients with tumors confined to submucosa (92.6 vs 42.8%, p < 0.001), more frequent presence of microvascular invasion (MVI) (3.6 vs 16.9%, p < 0.001) or peri-neural invasion (PNI) (2.0 vs 24.1%, p < 0.001). Incidence of lymph node and distant metastasis was 5.2 and 2.1% in G1 NET compared with 44.2 and 31.4% in G2 tumor, respectively (p < 0.001). For tumors sized 1–2 cm and confined to submucosa, incidence of lymph node metastasis was 6.1% for G1 NET compared with 21.1% for G2 NET. Status of MVI/PNI was predictive of lymph node metastasis for G2 tumor rather than G1 NET in this subgroup.

**Conclusions:**

Rectal G2 NET was much more invasive with significantly elevated prevalence of lymph node metastasis compared with G1 tumor.

## Introduction

Neuroendocrine tumor (NET) of the rectum includes three subgroups of tumors with great heterogeneity. According to mitotic count or Ki-67 index, NET is divided into three subgroups: well-differentiated G1 NET with indolent nature and favorable prognosis, moderately-differentiated G2 NET with intermediate risk of metastasis, and poorly-differentiated G3 NET (also termed as neuroendocrine carcinoma, NEC) with frequent metastasis and dismal outcome (1, 2). Evidence from the Surveillance, Epidemiology, and End Results (SEER) registries has indicated that the median survival for localized, regional, and distant disease is 223/111/33 months in well- and moderately-differentiated NET compared with 34/14/5 months in poorly-differentiated NET, respectively (3, 4). Grade is a dominant predictor for metastasis of rectal NET (5). Therefore, precise classification of tumor grade is important for management of rectal NET. However, due to low prevalence of rectal NET, diagnosis and evaluation of tumor grade is sometimes difficult in some hospitals without large sample size of patients. Information about tumor grade is frequently missing in most reported literature from nationwide or multi-center database (6, 7). Data from National Cancer Database of the America included 16,531 cases of rectal NET from 2004 to 2015, of which tumor grade was unknown in 59.9% of patients (4). Besides, most reports have included G1 NET and G2 NET together, termed as carcinoid. Since G1 tumor accounts for approximately 80–90% of rectal NET. This would underestimate the metastatic risk of this disease. Up to now, few studies have focused on detailed information about clinicopathologic feature, treatment modality and prognosis of rectal NETs based on different grades (G1/G2/G3). Direct comparison of rectal NET G1 with G2 tumor is necessary regarding more precise therapy.

Prediction of lymph node metastasis plays crucial role for management of rectal carcinoid according to consensus guidelines (1, 2). For rectal carcinoid sized smaller than 10 mm and confined to submucosa, local excision is suggested to be enough due to rare incidence of lymph node metastasis. A report enrolling 788 cases with T1 rectal carcinoid tumors from The Surveillance Epidemiology and End Results (SEER) database indicated that prevalence of metastasis was 1.1% for tumors ≤ 10 mm compared with 6.6% in lesions 11 to 19 mm (8). Another national cohort study from National Cancer Database (NCDB) enrolled 17,448 cases of rectal NET, of which 4.2% of cases were moderate-differentiated tumors (G2). The results indicated that prevalence of lymph node metastasis was 2.5% for tumors ≤ 10 mm compared with 12.8% for tumors sized 11–20 mm (4). By contrast, evidence from multi-institutional studies of European and North American centers (9) or Japan (7) indicated much higher prevalence of lymph node metastasis: 7–8% for tumors sized ≤10 mm and 31–40% for tumors sized 11–20 mm, respectively. For rectal carcinoid larger than 20 mm, prevalence of lymph node metastasis increased as high as 24.1–58% (4, 7). Therefore, radical resection with regional lymphadenectomy was recommended.

Treatment of rectal carcinoid tumors sized 10 to 20 mm is still controversial. For patients with high risk of lymph node metastasis including presence of lymphovascular invasion (LVI) or peri-neural invasion (PNI), radical resection with regional lymphadenectomy is recommended. Data from NCDB of the America indicated that about three quarters (755/1,013, 263/342) of rectal carcinoid sized 11–20 mm or 10–20 mm received local excision (4, 10). By contrast, a nationwide cohort in Japan from 1984 to 1998 enrolled 345 cases of colorectal carcinoids (rectum: 92%), of which only 19% of cases received endoscopic resection and 80% of cases received surgery (7). Several reasons might contribute to the difference between western and eastern countries. First, percentage of cases with tumors smaller than 10 mm was 79.8% in the cohort from NCDB of the America compared with 63% in the cohort from Japan. Second, the cohort from Japan was in the earlier era when endoscopic resection was not widely used. More importantly, moderately differentiated G2 NET consisted only 4.2% of the cohort from NCDB, which was much lower than that reported from other countries (11). Information about tumor differentiation grade was unknown in 59.9% of cases from NCDB cohort and not mentioned in the Japan cohort. Due to frequent loss of information about tumor grade (12), comparison of results from different institutions seemed difficult. Comparison of G1 with G2 rectal NET is therefore necessary for better understanding of the disease and optimal choice of treatment.

In the present study, we analyzed 601 cases of rectal G1 and G2 NETs diagnosed in our center. Our results demonstrated that, compared with G1 NET, G2 tumors were with significantly larger tumor size, deeper invading depth, more frequent presence of microvascular invasion or peri-neural invasion, which were associated with elevated incidence of lymph node metastasis and distant metastasis. For tumors sized 1–2 cm and confined to submucosa, local excision might be appropriate, for which evaluation of MVI/PNI was useless. By contrast, for G2 tumors, radical resection was recommended especially for those with presence of MVI/PNI. Our results would help discriminate the metastatic potential as well as treatment modalities for indolent G1 NET compared with moderately-invasive G2 tumor.

## Materials and Methods

**Figure 1:**
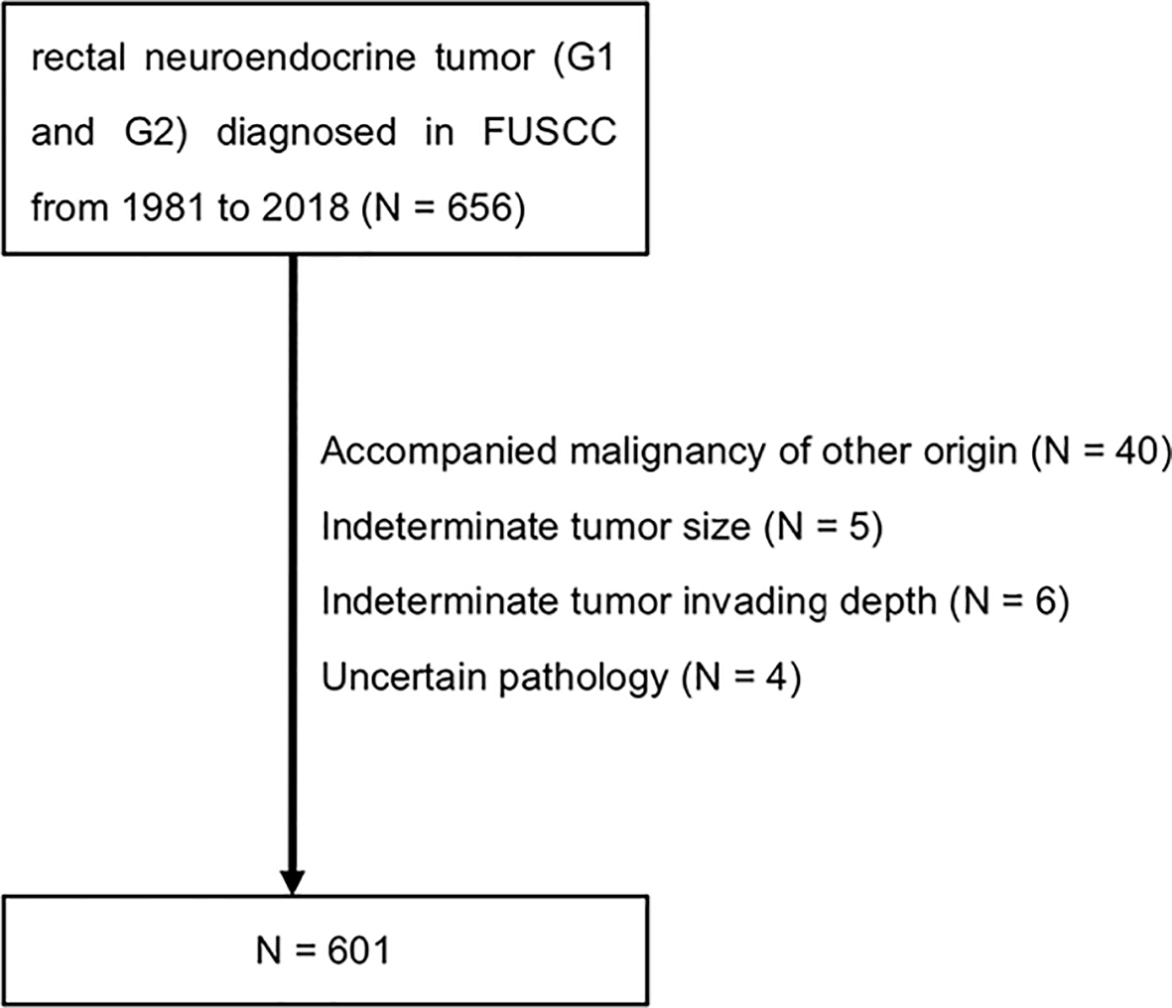
Flowchart of the study population. FUSCC, Fudan University Shanghai Cancer Center.

From 1981 to 2018, 656 cases of rectal neuroendocrine tumors (NET) were diagnosed and treated in Shanghai Cancer Center Fudan University (FUSCC). 55 cases were excluded from analysis, of which 40 cases were with accompanied malignancy of other origin, 5 cases were with indeterminate tumor size, 6 cases were with unknown tumor invading depth and 4 cases were with uncertain pathology (**Figure 1**). All the cases were pathologically confirmed. Tumor grade was determined by cell mitoses or Ki-67 index (2) as well as histology: <2 mitoses/HPF or <3% Ki-67 index for G1, 2-20 mitoses/HPF or 3–20% Ki-67 index for G2, respectively (13). Tumor staging was conducted according to European Neuroendocrine Tumor Society (ENETS) TNM classification for NET of the colon and rectum (2), in which T stage was combination of tumor invading depth and tumor size. Tumors invaded mucosa or submucosa and sized ≤ 2 cm were defined as stage T1, tumors invaded muscularis propria or sized >2 cm were defined as stage T2. Clinicopathologic data was recorded from hospital database. This study was approved by the Research Ethics Committee of FUSCC. Informed consent was obtained from all participants. The last follow-up time was June 2019. Data analysis was performed using IBM SPSS statistics version 23. Chi-square analysis was used to test differences among subgroups. A two-sided p < 0.05 was considered as statistically significant.

## Results

### Clinicopathologic Feature of the Cohort

Of 601 cases of rectal neuroendocrine tumors (**Table 1**), 346 (57.6%) cases were male. Median values for patient age, distance from anal verge and tumor diameter were 50 years old (range 18–83), 5 cm (range 1–15) and 0.7 cm (range 0.2–13.0), respectively. 515 (85.7%) cases were with grade G1 and 86 (14.3%) cases were with grade G2. 513 (85.4%) cases were with tumors confined to submucosa and 86 (14.3%) cases were with tumors invading deeper than muscularis propria. Sixty-five (10.8%) cases were with regional lymph node metastasis and 38 (6.3%) cases were with distant metastasis. Percentages of patients with ENETS TNM stage I, II, III, and IV were 83.2, 3.5, 7.0, and 6.3%, respectively. For evaluation of microvascular invasion (MVI), information was unknown for 289 (48.1%) cases. 19 patients were with presence of MVI and 293 cases were with absence of MVI. For evaluation of perineural invasion (PNI), information was indeterminate for 294 (48.9%) cases. Nineteen cases were with positive PNI and 288 cases were with negative PNI. Thirteen (2.1%) patients gave up for any treatment, 511 (85.0%) cases received local excision and 77 (12.8%) cases received radical resection.

**Table 1:** Clinicopathologic Features of 601 cases of G1/G2 rectal NET.

**Gender**	Male	346 (57.6%)
	Female	255 (42.4%)
**Age (year)**	Median 50 (18–83)	
**Distance from anal verge (cm)**	Median 5 (1–15)	
**Tumor size (cm)**	Median 0.7 (0.2-13.0)	
**Tumor grade**	G1	515 (85.7%)
	G2	86 (14.3%)
**Invading depth**	T0	399 (66.4%)
	T1	114 (19.0%)
	T2	34 (5.7%)
	T3	31 (5.2%)
	T4	21 (3.5%)
**N stage**	N0	536 (89.2%)
	N1	65 (10.8%)
**M stage**	M0	563 (93.7%)
	M1	38 (6.3%)
**ENETS TNM stage**	Ia	392 (65.2%)
	Ib	108 (18.0%)
	II	21 (3.5%)
	III	42 (7.0%)
	IV	38 (6.3%)
**Microvascular invasion**	no	293 (48.8%)
	yes	19 (3.2%)
	unknown	289 (48.1%)
**Perineural invasion**	no	288 (47.9%)
	yes	19 (3.2%)
	unknown	294 (48.9%)
**Surgical modality**	none	13 (2.1%)
	EMR	49 (8.2%)
	ESD	196 (32.6%)
	TEM	266 (44.3%)
	AR/APR/hartman	77 (12.8%)

EMR, Endoscopic Mucosal Resection; ESD, Endoscopic Submucosal Dissection; TEM, Transanal Endoscopic Microsurgery; AR, Anterior Resection; APR, Abdomen Perineal Resection.

### Comparison of Rectal G1 NET With G2 Tumor

Using chi-square analysis, we tested the difference between rectal G1 NET with G2 tumor (**Table 2**). Distribution of patient gender (p = 0.290) as well as distance from anal verge (p = 0.768) was not significantly different between G1 and G2 NETs. Patients diagnosed with G1 NET were younger than patients with G2 disease (49 vs 52 years old, p = 0.043). Compared with G1 NET, patients with G2 tumor were with significantly larger tumor size (0.8 vs 2.2 cm, p = 3.3E-10), less percentages of cases with tumor confined to submucosa (92.6 vs 42.8%, p = 4.5E-47), elevated incidence of lymph node metastasis (5.2 vs 44.2%, p = 5.0E-27) as well as distant metastasis (2.1 vs 31.4%, p = 5.6E-25). Percentages of patients with ENETS TNM stage I disease were much fewer for patients with G2 NET compared with G1 tumor (36.0 vs 91.1%, p = 3.2E-39). Presence of microvascular invasion (16.9 vs 3.6%, p = 1.0E-4) or perineural invasion (24.1 vs 2.0%, p = 2.9E-10) was much more common for G2 NET compared with G1 disease. The 92.9% of cases received local excision for G1 NET, compared with 48.1% for G2 tumors (p = 1.6E-27).

**Table 2:** Comparison of rectal G1 NET with G2 tumor (n = 601).

		G1	G2	p
Gender	male	292	54	0.290
	female	223	32	
Age	mean	49	52	0.043
Distance from anal verge (cm)	mean	5.9	5.8	0.768
Tumor size (cm)	mean	0.8	2.2	3.3E-10
Invading depth*	T0	394	5	4.5E-47
	T1	83	31	
	T2	22	12	
	T3	6	25	
	T4	10	11	
	T0-1/total, %	92.6	42.8	
**N stage**	N0	488	48	5.0E-27
	N1	27	38	
	N1/total, %	5.2	44.2	
**M stage**	M0	504	59	5.6E-25
	M1	11	27	
	M1/total, %	2.1	31.4	
TNM stage**	Ia	376	16	3.2E-39
	Ib	93	15	
	II	15	6	
	III	20	22	
	IV	11	27	
	I/total, %	91.1	36.0	
Microvascular invasion***	no	244	49	1.0E-4
	yes	9	10	
	yes/total	3.6	16.9	
Perineural invasion***	no	244	44	2.9E-10
	yes	5	14	
	yes/total, %	2.0	24.1	
Surgical modality****	EMR	48	1	1.6E-27
	ESD	179	17	
	TEM	246	20	
	AR/APR/hartman	36	41	
	local excision/total, %	92.9	48.1	

EMR, Endoscopic Mucosal Resection; ESD, Endoscopic Submucosal Dissection; TEM, Transanal Endoscopic Microsurgery; AR, Anterior Resection; APR, Abdomen Perineal Resection.

*T stage only indicates tumor invading depth without consideration of tumor diameter.

**TNM stage is according to ENETS TNM staging system, in which T stage is determined by combination of invading depth and tumor size.

***Information about microvascular invasion and perineural invasion is lost for 48.1% and 48.9% of cases.

****Local excision includes the sum of EMR, ESD, and TEM.

### Risk Factors Predicting Lymph Node Metastasis for Rectal NET

Management of rectal NET was decided by predicted risk of regional lymph node metastasis, which was mainly influenced by tumor size, invading depth and MVI/PNI positivity. For tumors sized <1cm, 1–2 cm and >2 cm subgroups, incidence of lymph node metastasis was 0.5, 11.6, and 57.1% for G1 NET (p = 3.7E-31) compared with 0, 43.2, and 68.8% for G2 NET (P = 2.4E-5), respectively. For tumors with invading depth of T0-1, T2, T3, and T4 subgroups, risk of lymph node metastasis was 1.4, 36.4, 66.7, and 80.0% for G1 NET (p = 2.9E-46) compared with 11.1, 58.3, 76.0, and 63.6% for G2 NET (p = 2.0E-6). Compared with patients without microvascular invasion (MVI), patients with presence of MVI were with higher incidence of lymph node metastasis for G1 NET (7.4 vs 33.3%, p = 0.030) as well as for G2 NET (42.9 vs 80.0%, p = 0.042). For G1 NET, presence of perineural invasion (PNI) was not associated with elevated risk of lymph node metastasis (p = 0.359). Incidence of lymph node metastasis was significantly increased for G2 NET with presence of PNI compared with G2 tumor without presence of PNI (92.9 vs 34.1%, p = 1.3E-4).

Taking tumor size and invading depth together as recommended by consensus guideline for management of rectal NET, we further divided the cohort into three subgroups: tumors smaller than 1 cm and confined to submucosa, tumors sized 1–2 cm and confined to submucosa, tumor larger than 2 cm or invading deeper than muscularis propria. Incidence of lymph node metastasis in three subgroups was 0.3, 6.1, and 51.3% for G1 NET compared with 0, 21.1, and 68.8% for G2 NET, respectively. Of 149 cases with tumors sized 1–2 cm, 83 cases were with complete information of MVI and PNI for analysis. Presence of MVI/PNI was not significantly associated with increased risk of lymph node metastasis for G1 tumor (p = 0.546). For G2 tumor, presence of MVI/PNI was significantly associated with elevated incidence of lymph node metastasis (25.0 vs 100%, p = 0.014).

### Treatment Modalities for Subgroups According to Consensus Guideline

We analyzed treatment modalities for subgroups according to consensus guideline for management of rectal NET (**Table 4**). Thirteen cases receiving no surgery were excluded from analysis. For patients with rectal G1 NET (n = 509), incidence of lymph node metastasis was 0.3% for tumors sized <1 cm and confined to submucosa, 98.1% of patients received local excision. For tumors sized >2 cm or invading through muscularis propria, incidence of lymph node metastasis was 55.9 and 61.8% of patients received radical resection. For tumors sized 1–2 cm and confined to submucosa, lymph node metastasis occurred in 8.2% of patients with negative microvascular invasion (MVI) or perineural invasion (PNI), of which 85.7% of patients received local excision. By contrast, no patient suffered from lymph node metastasis for patients with positive MVI or PNI, of which none received radical resection. Of 49 patients with unknown MVI/PNI status, lymph node metastasis rate was 4.1 and 98.0% (48/49) of patients received local excision.

For patients with G2 NET, 16 patients were with tumors sized <1 cm and confined to submucosa, of which incidence of lymph node metastasis was 0% and all the patients received local excision. 43 patients were with tumors larger than 2 cm or invading deeper than muscularis propria, of which lymph node metastasis rate was 72.1 and 86.0% of patients received radical resection. Of 19 patients with tumors sized 1–2 cm and confined to submucosa, 11 patients were with negative MVI/PNI. In 18.2% of patients, lymph node metastasis occurred and 90.9% of patients received local excision. One patient was with positive MVI/PNI, which suffered from lymph node metastasis and thus received radical resection. Information about MVI/PNI was unknown for seven patients, of which one patient suffered from lymph node metastasis and thus received radical resection.

Taking G1 and G2 NETs together into consideration, 392 patients were with tumors smaller than 1 cm and confined to submucosa, of which one patient suffered from lymph node metastasis and seven patients received radical resection. Seventy-seven patients were with tumors larger than 2 cm or invading deeper than muscularis propria, of which 50 patients suffered from lymph node metastasis and 58 patients received radical resection. One hundred-eighteen patients were with tumors sized 1–2 cm and confined to submucosa, of which 60 patients were with negative MVI/PNI. Six patients suffered from lymph node metastasis and eight patients received radical resection. Two patients were with positive MVI or PNI, of which one patient suffered from lymph node metastasis and received radical resection. Of 56 patients with unknown MVI/PNI status, three patients suffered from lymph node metastasis and two patients received radical resection.

## Discussion

Annual incidence of neuroendocrine tumor (NET) is steadily increasing from 1.09/100,000 (1973) to 5.25/100,000 (2004) in the United States, of which the rectum was the most common primary site in Asian/pacific Islander (3). A nation-wide retrospective epidemiological survey from 23 hospitals in China has also demonstrated a significantly increased incidence of gastroenteropancreatic NET from 2001 to 2010 and pancreas (31.5%) and the rectum (29.6%) are the most common primary sites (14). Most reported literature has enrolled rectal NET with G1 grade and G2 grade together as carcinoid, probably due to rare incidence of G2 tumor. Current consensus guideline for management of rectal carcinoid also takes indolent G1 tumor with moderately-invasive G2 tumor together into consideration. However, rectal G2 NET is with higher metastatic potential and poorer prognosis compared with G1 NET (11). Comparison of rectal G1 NET with G2 NET is necessary for more précising therapy of this disease.

Rectal NET grade G1 and grade G2 are with different metastatic potential and prognosis, of which the 5-year survival is 97.7 and 60.0%, respectively (11). However, G1 and G2 tumors are frequently included together for analysis in most reported literature. Information about tumor differentiation grade is commonly lost in majorities of reports. A report from National Cancer Database of the America including 17,448 cases of rectal NET indicated that tumor grade was unknown for 59.9% of patients (4). Therefore, comparison of results from different institutions will be difficult due to varied percentages that G1/G2 tumors accounted for. In our study, G2 tumors exhibited larger tumor size and deeper invading depth at diagnosis. The 92.6% of tumors were confined to submucora for G1 tumor compared with 42.8% for G2 tumor. The 44.2% of patients with G2 NET were with lymph node metastasis (**Table 2**), which means about half of G2 tumors can’t be locally resected. By contrast, local excision is appropriate for approximately 80–90% of rectal G1 NET, as demonstrated by evidence from nation-wide database indicating that percentages of rectal NET receiving local excision is 79.4, 80, and 92.4% in America, Japan (7), and Korea (6), respectively.

Risk of lymph node metastasis is the most important determinant for deciding whether to receive local excision or radical resection, which was reported to be varied a lot from different countries. Evidence from national database indicated that prevalence of lymph node metastasis for rectal NET is 5.0% in Korea (6), 12.8% in America (6), and 15.1–31.0% in Japan (7, 15), respectively. In our study, prevalence of lymph node metastasis at initial presentation was 44.2% for G2 tumor compared with 5.2% for G1 tumor. Metastatic risk for G2 tumor was much higher than that in above-mentioned literature enrolling mixed population of G1 and G2 tumors together. Indeed, Juha Jernman reported that metastatic risk for G1, G2, and G3 rectal NETs were 0, 81.8, and 100%, respectively (16). In a cohort of 98 patients with rectal NET, diminutive tumor (< 1 cm) that metastasized were all G2 (5). Local excision should be done only in carefully selected patients with G2 rectal NET.

Management of rectal NET sized 1–2 cm is complicated. Patients with presence of microvascular invasion (MVI) or peri-neural invasion (PNI) are suggested to receive radical resection (1, 17). In our study, incidence of lymph node metastasis was 11.6% for G1 tumors sized 1–2 cm and 6.1% for G1 tumors sized 1–2 cm and confined to submucosa. Status of MVI/PNI was not predicted for lymph node metastasis risk for G1 tumors sized 1–2 cm and confined to submucosa (p = 0.546, **Table 3**). The 91.9% (91/99) of patients received local excision in our study (**Table 4**). Therefore, local excision might be enough for G1 tumors sized 1–2 cm and confined to submucosa. For G2 tumors, incidence of lymph node metastasis was 43.2% for tumors sized 1–2 cm and 21.1% for tumors sized 1–2 cm and confined to submucosa (**Table 3**). Presence of MVI or PNI was significantly associated with increased risk of lymph node metastasis. Therefore, local excision could only be done for carefully selected patients in this subgroup. Radical resection should be performed for patients with presence of MVI/PNI. What should be mentioned, information about MVI/PNI was frequently lost in reported literature. A multicenter study from Korea indicated that information about lymphovascular invasion was indeterminate for 43.2% of rectal NETs (18). In our study, information about MVI or PNI was unknown for 48.1 and 48.9% of patients (**Table 1**). Evaluation of MVI/PNI should be routinely done in clinical practice, which is especially important for rectal NETs sized 1–2 cm.

**Table 3:** Risk factors associated with lymph node metastasis for rectal NET.

	G1 n = 515			G2 n = 86			G1+G2 n = 601		
metastasis	no	yes	%*	no	yes	%	no	yes	%
Tumor size (cm)	p = 3.7E-31			p = 2.4E-5			p = 9.8E-47		
<1	380	2	0.5	17	0	0	397	2	0.5
1-2	99	13	11.6	21	16	43.2	120	29	19.5
>2	9	12	57.1	10	22	68.8	19	34	64.1
Invading depth**	p = 2.9E-46			p = 2.0E-6			p = 7.8E-63		
T0-1	470	7	1.4	32	4	11.1	502	11	2.1
T2	14	8	36.4	5	7	58.3	19	15	44.1
T3	2	4	66.7	6	19	76.0	8	23	74.2
T4	2	8	80.0	4	7	63.6	6	15	71.4
MVI	p = 0.030			p = 0.042			p = 1.8E-5		
no	226	18	7.4	28	21	42.9	254	39	13.3
yes	6	3	33.3	2	8	80.0	8	11	57.9
PNI	p = 0.359			p = 1.3E-4			p = 7.7E-9		
no	224	20	8.2	29	15	34.1	253	35	12.1
yes	4	1	20.0	1	13	92.9	5	14	73.7
Size and depth	p = 5.7E-41			p = 4.2E-7			p = 1.4E-60		
<1cm and T1	376	1	0.3	17	0	0	393	1	0.3
1-2cm and T1	93	6	6.1	15	4	21.1	108	10	8.5
>2cm or T2	19	20	51.3	15	33	68.8	34	53	60.9
1-2cm and T1 subgroup***	p = 0.546			p = 0.014			p = 0.014		
MVI/PNI -	49	10	16.9	12	4	25.0	61	14	18.7
MVI/PNI +	3	1	25.0	0	4	100	3	5	62.5

MVI, Micro-Vascular Invasion; PNI, Peri-Neural Invasion.

Chi-square analysis was used.

*% = yes/(yes + no).

**T stage only indicates tumor invading depth, without consideration of tumor size.

***One hundred-eighteen cases were with tumors sized 1-2 cm in diameter and confined to submucosa, of which 83 cases were with complete information of MVI and PNI for analysis.

**Table 4:** Treatment modalities according to consensus guideline (n = 588).

Grade	Depth and size	MVI/PNI	LNM	Total	Local excision	Radical resection
G1	T0-1 and <1cm	/	1(0.3%)	376	369(98.1%)	7
	T0-1 and 1-2cm	–	4(8.2%)	49	42(85.7%)	7
		+	0(0%)	1	1	0(0%)
		unknown	2(4.1%)	49	48	1
	>2cm or T2-4	/	19(55.9%)	34	13	21(61.8%)
G2	T0-1 and <1cm	/	0(0%)	16	16(100%)	0
	T0-1 and 1-2cm	–	2(18.2%)	11	10(90.9%)	1
		+	1(100%)	1	0	1(100%)
		unknown	1(14.3%)	7	6	1
	>2cm or T2-4	/	31(72.1%)	43	6	37(86.0%)
G1+G2	T0-1 and <1cm	/	1(0.3%)	392	385(98.2%)	7
	T0-1 and 1-2cm	–	6(10.0%)	60	52(86.7%)	8
		+	1(50.0%)	2	1	1(50.0%)
		unknown	3(5.4%)	56	54	2
	>2cm or T2-4	/	50(64.9%)	77	19	58(75.3%)

MVI, Micro-Vascular Invasion; PNI, Peri-Neural Invasion, LNM, Lymph Node Metastasis.

Chi-square analysis was used.

Thirteen cases receiving no surgery were excluded from analysis.

## Data Availability Statement

The raw data supporting the conclusions of this article will be made available by the authors, without undue reservation.

## Ethics Statement

The studies involving human participants were reviewed and approved by the Ethical Committee of FUSCC (no.: 050432-4-1212B). The patients/participants provided their written informed consent to participate in this study.

## Author Contributions

Conception and design: Y-WL, Y-PH, and M-HW. Provision of study materials and patients: M-HW, YX, and S-JC. Data collection: J-JP and F-QL. Data analysis and interpretation: Y-WL and Y-PH. All authors contributed to the article and approved the submitted version.

## Funding

This study was supported by National Natural Science Foundation of China (No. 81301761 and 81372646) and Research Fund for the Doctoral Program of Higher Education of China (No. 20130071120070).

## Conflict of Interest

The authors declare that the research was conducted in the absence of any commercial or financial relationships that could be construed as a potential conflict of interest.
